# Fixation stability of the upward gaze in patients with myasthenia gravis: an eye-tracker study

**DOI:** 10.1136/bmjophth-2017-000072

**Published:** 2017-11-16

**Authors:** Miharu Mihara, Atsushi Hayashi, Kazuya Fujita, Ken Kakeue, Ryoi Tamura

**Affiliations:** 1 Department of Ophthalmology, Graduate School of Medicine and Pharmaceutical Sciences, University of Toyama, Toyama, Japan; 2 Department of Integrative Neuroscience, Graduate School of Medicine and Pharmaceutical Sciences, University of Toyama, Toyama, Japan

**Keywords:** diagnostic tests/investigation, muscles, physiology

## Abstract

**Objective:**

To quantify fixation stability of the upward gaze in patients with myasthenia gravis (MG) using an eye tracker.

**Methods and analysis:**

In this study, 21 normal subjects, 5 patients with MG with diplopia, 5 patients with MG without diplopia and 6 patients with superior oblique (SO) palsy were included. Subjects fixated on a target in the upward direction for 1 min. The horizontal (X) and vertical (Y) eye positions were recorded using an eye tracker. Fixation stability was first quantified using the bivariate contour ellipse areas (BCEA) of fixation points as an index of whole stability. Then, the SDs of the X and Y eye positions (SDX and SDY, respectively) were quantified as indices of directional stability, with the data divided into three 20 s fractions to detect temporal fixation fluctuation.

**Results:**

BCEAs were larger in patients with MG (both with and without diplopia) than normal subjects and patients with SO palsy, without significant differences among the three 20 s fractions. Compared with normal subjects, SDXs were larger only in patients with MG with diplopia; SDYs were larger in both patients with MG with and without diplopia. In addition, SDYs in patients with MG with diplopia were larger than those in patients with MG without diplopia and patients with SO palsy. Furthermore, a significant difference among the three 20 s fractions was detected for SDYs in patients with MG with diplopia.

**Conclusion:**

Patients with MG, especially those with diplopia, exhibit fixation instability in the upward gaze. Non-invasive quantification of fixation stability with an eye tracker is useful for precisely identifying MG-specific fatigue characteristics.

**Trial registration number:**

UMIN000023468; pre-results.

**Key messages**

**What is already known about this subject?**

Subjects with myasthenia gravis (MG) often present with ocular symptoms, including diplopia and ptosis. Because these symptoms greatly impact patient quality of life, knowing how treatments affect them is important. Current clinical evaluations of MG diplopia are subjective and poor quantification of variability caused by MG fatigability, which make it difficult to study diplopia and evaluate treatment efficacy.

**What are the new findings?**

Eye-tracking systems can be used to objectively quantify fixation stability. Using these measurements, it was found that fixation of upward gaze was less stable in the vertical eye position than in the horizontal eye position. Additionally, MG subjects with diplopia have worse fixation stability than both normal subjects and those with a superior oblique palsy. Interestingly, fluctuation in upward gaze fixation seemed to decrease over time in patients with MG.

**How might these results change the focus of research or clinical practice?**

Gaze instability caused by fatigability associated with MG can be more accurately evaluated by quantifying fixation with eye tracking and considering how fixation changes over time.

## Introduction

Approximately 60% of patients with myasthenia gravis (MG) have diplopia at the time of diagnosis.^1^ Therefore, the majority of patients with MG with diplopia are first examined by an ophthalmologist. Zambelis *et al* 2 reported that 38% of patients with isolated ptosis and/or diplopia who are referred for electrophysiological evaluation have MG. Furthermore, they stated that the presence of both diplopia and ptosis was more likely due to MG than any other disease. If MG is suspected, additional examinations are needed to obtain a definitive MG diagnosis. These examinations included ptosis evaluation before and after an edrophonium test and an ice pack test.^3^ Additionally, the presence of acetylcholine receptor antibodies or muscle-specific receptor tyrosine kinase antibodies in the serum, ‘waning’ during repetitive nerve stimulation and ‘increased jitter’ on a single-fibre electromyography can confirm the presence of MG.^3^

A quantitative MG score and an MG composite scale are used to evaluate disease severity. For these measures, diplopia is evaluated by asking the patient to gaze in one direction. The time from gaze to subjectively determined diplopia appearance is measured.^4 5^ The 1 min upper gaze test is used to identify ptosis and diplopia and to determine whether the MG fatigue phenomenon is present.^6^ However, these evaluations are subjective with the patient condition determined using physician observation or patient symptom reporting. In clinical observation, ophthalmologists infer the presence of diplopia and its severity by measuring ocular alignment and motion using the alternate prism cover test (APCT) and the Hess screen test, respectively.^7^ These tests, however, are not objective and have poor reproducibility in patients with MG because ocular alignment is quantified using only one data point (ocular position). This is problematic because ocular alignment and range of ocular motion change over time in patients with MG^7^ because their muscles easily fatigue which is a characteristic of MG. Therefore, objective and reproducible evaluations of gaze instability caused by fatigability in patients with MG are needed and should include sequential eye position data obtained with a reasonably high sampling frequency (more than 200 Hz).^8–10^ There are several studies that have examined saccadic eye movements of patients with MG using electro-oculography or infrared scleral reflection or scleral search coil^11–14^ and reported that the peak velocity of saccade in patients with MG was similar to or greater than that of normal subjects.^11 12^ These studies, however, did not evaluate gaze stability of patients with MG by measuring fixation position, although fixation methods are more widely used and meaningful clinical tests (eg, the Quantitative MG score, MG composite scale) for the gaze stability of patients with MG.

An eye tracker is capable of measuring ocular position continuously for a long enough period with relatively high time resolution. Upward is the most sensitive gaze direction for detecting the ocular MG symptoms (ie, diplopia and ptosis) because extraocular muscle weakness is known to appear most prominently in the elevator muscles of patients with MG.^6 15 16^ Therefore, in the present study, fixation stability in upward gaze was quantified by using an eye tracker in subjects with and without MG.

## Materials and methods

All study subjects provided written informed consent to participate in the study and all study conduct adhered to the tenets of the Declaration of Helsinki.

### Participants

Four groups of volunteers (normal subjects, patients with MG with diplopia, patients with MG without diplopia and patients with superior oblique (SO) palsy) were recruited for the study; the SO palsy group was included to determine whether the presence of diplopia itself affects the fixation stability. The diagnosis of MG was made based on clinical (fluctuating symptoms with easy fatigability and recovery after rest), immunological and neurophysiological findings.^3^ Diagnostic tests included anti-acetylcholine receptor antibodies, anti-muscle-specific tyrosine kinase antibodies, edrophonium test and decremental muscle response to a train of low-frequency repetitive nerve stimuli. Participants in all groups did not have dyscoria and could clearly watch the target when vision was uncorrected or corrected using soft contact lenses. Patients whose best corrected decimal visual acuity was less than 1.0 (Snellen: 20/20) were not included because of the possible disturbance in visual fixation caused by lowered visual acuity. Patients were not excluded because of age, MG type or disease duration, except for those who could not fixate the target of upward from the beginning.

### Set-up for eye position monitoring

**Figure 1:**
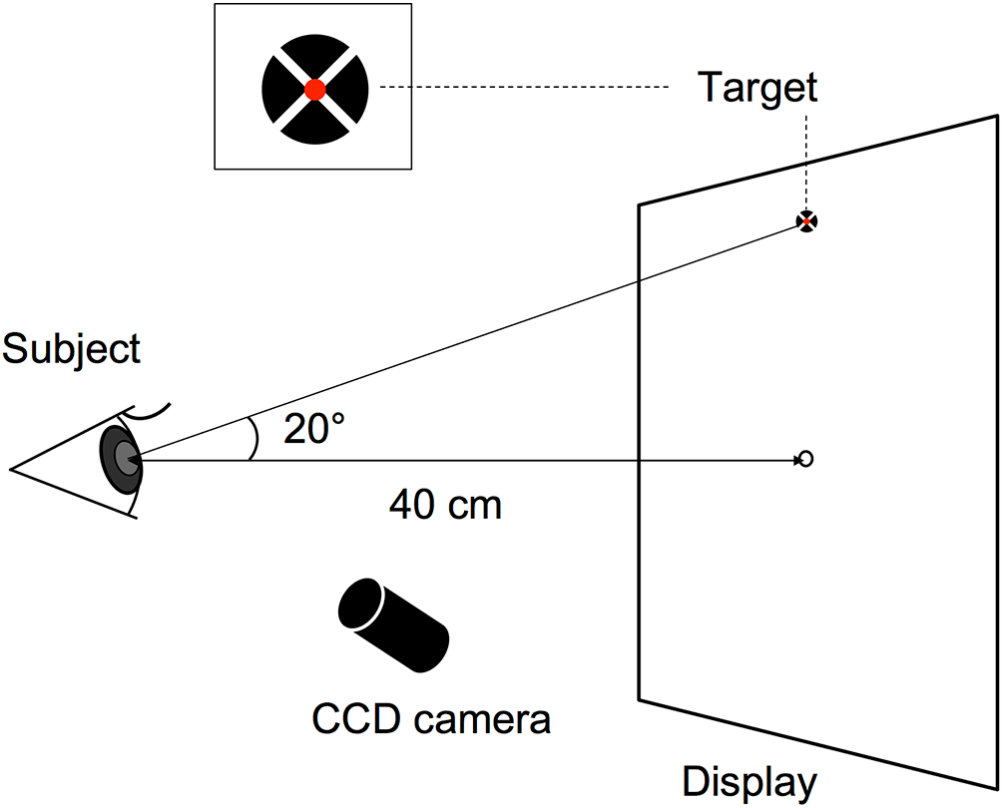
The fixation target was positioned 20° above each subject’s horizontal plane (primary position) to induce an upward gaze and was displayed on a computer monitor at a distance of 40 cm. CCD, charge-coupled device.

The horizontal (X) and vertical (Y) positions of both eyes were recorded using the ViewPoint EyeTracker system (Arrington Research, Scottsdale, AZ, USA) at a sampling rate of 220 Hz, as was previously done by Crossland *et al*.^8–11^ The ViewPoint system consists of a display for visual stimulation (Diamondcrysta, RDT222WM-S, Mitsubishi, Tokyo, Japan, resolution was 1680×1050 pixels, and refresh rate was 60 Hz) and two infrared cameras that were mounted on the head positioner. Each subject sat in a chair facing the display set at a distance of 40 cm from the subject, with horizontal eye level even with the centre of the display, the primary position (figure 1). The subject’s head was stabilised against a head and chin rest. The cameras sent images of the eye to a computer via a USB cable. The eye tracker determined eye position using the dark pupil technique and eye-tracking data were stored on a hard disk for offline data analysis. Prior to the fixation stability testing described in the next section, a 16-point grid (4×4 matrix) calibration and a subsequent validation procedure was performed for each subject using software supplied by the eye-tracker manufacturer (ViewPoint EyeTracker Software User Guide).

### Assessing fixation stability

The fixation target consisted of a red inner circle (0.2° in diameter) superimposed at the centre of a black circle (1.5° in diameter) and a white cross was located in the centre of the circular target (figure 1), as previously reported by Thaler *et al*.^17^ The target was positioned 20° above each subject’s horizontal plane (primary position) to induce an upward gaze and was displayed on a computer monitor. Subjects were instructed to keep their gaze fixed on the target for 1 min with both eyes. During examinations, patients with ptosis were examined with their eyelids mildly lifted using a surgical tape (to the extent that eyes did not dry) so that the eye tracker could detect the pupil of the subject even when gazing at 20° upward, and therefore, the eyelid descent itself was not a problem.

### Data analysis and statistical methods

Data obtained immediately after initiating target presentation were discarded because these data included saccadic eye movements related to beginning fixations. As this period of eye movements varied from subject to subject, the data that accompanied the eye movements were discarded manually by visual inspection of the eye position chart in each subject. Data that contained artefact noise, related to blinking or erroneous pupil image capture, were also excluded after manual confirmation of artefact presence using stored data. For blinks and erroneous pupil image capture, the data obtained 100 ms before and after the onset of these events were removed from analysis.^18^ Data were selected on the dominant eye in all patients. The dominant eye was determined using the Hole-in-Card test.^19^ Approximately 5% of the overall data were excluded due to blinks and noisy recording.

Fixation stability was first quantified using bivariate contour ellipse areas (BCEA) encompassing fixation points as an index of whole fixation stability, with the data divided into three 20 s fractions (ie, 0–20 s, 20–40 s and 40–60 s).

The BCEA was calculated using the following formula^8^:

(1) $$BCEA=2k\pi \sigma_{H}\sigma_{V}(1-\rho^{2}{)}^{1/2}$$

where σ_H_ and σ_V_ are the SDs of the horizontal and vertical eye positions, respectively, and ρ is their Pearson product-moment correlation. The *k* is dependent on the probability area chosen and is calculated using the following equation:

(2) $$P=1-e^{-k}$$

where *e* is the base of the natural logarithm. For this study, fixation data were calculated using P values of 0.68 (ie, 1 SD), which led to *k* values of 1.14.

Then the SDs of the X and Y eye positions were calculated as indices of detailed (individual direction) stability with the data divided into the three 20 s fractions. Although BCEA and SDs are not independent measures, both parameters were used, because BCEA is presently one of the standard indexes for evaluation of whole fixation stability as described in previous studies.^8 10 20^

A repeated measures analysis of variance (ANOVA) was performed for the data on 68% BCEA and SDs with groups as a between-subjects variable and the 20 s fractions as a within-subjects variable. If a significant difference was identified, pairwise comparisons were performed using a two-tailed t-test with Bonferroni correction. All statistical analyses were performed using the JMP Pro software package (V.11.2.0, SAS Institute, Cary, NC, USA). P value less than 0.05 was considered statistically significant.

## Results

### Study subjects

A total of 21 normal, healthy subjects (mean age=48.9±18.6 years, nine women) who had no strabismus or oculomotor abnormalities were included in the study as a control group. The MG group consisted of 10 patients with MG (mean age=54.7±16.5 years, six women) with diplopia (n=5) and without diplopia (n=5) at disease presentation. These patients were diagnosed with MG based on the presence of antibodies in their serum (eight patients were acetylcholine receptor antibody positive, one was muscle-specific kinase antibody positive and one was seronegative), edrophonium test results (eight of nine patients tested positive and one patient was not tested) and repetitive nerve stimulation results (five of eight patients tested positive and two patients were not tested). The primary ocular deviations of the patients with MG without diplopia measured by APCT were orthophoria or slightly exophoria. In the patients with MG with diplopia, two patients exhibited exotropia: one was 45 prism dioptres (PD) exotropic, the other was both 20 PD exotropic and 3 PD left hypertrophic. Another patient exhibited 14 PD right hypertropia, one exhibited 16 PD exophoria and one was orthophoric but presented with disturbances of adduction and abduction in his right eye. The SO palsy group consisted of six patients with SO palsy with diplopia (mean age=53.5±16.3 years, four women). Patients with SO palsy in this study were unilateral palsy. This group consisted of patients with acquired SO palsy and patients with decompensated congenital SO palsy. They had awareness of diplopia in the upward gaze. Their ocular deviation measured by the synoptophore in the upward gaze was 3.5±5.22° of the horizontal deviation, 4.83±5.24° of the vertical deviation and 3.17±3.44° of the cyclodeviation.

There were no statistically significant differences in subject ages among groups (ANOVA; *F*_(3, 33)_=0.4, P=0.75).

### Upward gaze stability based on the measure of BCEAs

**Figure 2:**
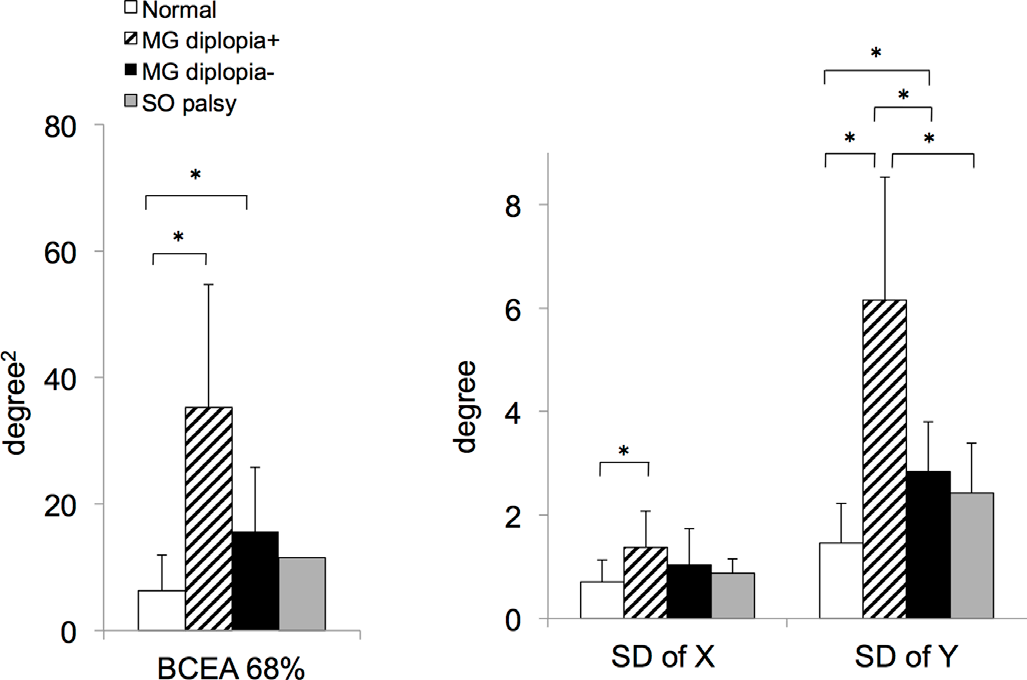
Results of 1 min fixation stability testing for normal subjects (white bars), patients with myasthenia gravis (MG) with diplopia (hatched bars), patients with MG without diplopia (black bars) and patients with superior oblique (SO) palsy (grey bars). The 68% bivariate contour ellipse area (BCEA) was significantly larger in the MG with diplopia and MG without diplopia groups than in the normal group (P<0.05). The SD of X position was significantly larger in the MG with diplopia group than in the normal group (P<0.05). The SD of Y position was significantly larger in the MG with diplopia group than other groups and in the MG without diplopia group than in the normal group (P<0.05). Means are shown and error bars indicate SDs. *Indicates P<0.05.

A repeated measures ANOVA test revealed that there was a significant main effect of study groups for the 68% BCEA (*F*_(3, 33)_=1.46, P<0.0001). The 68% BCEA was significantly larger in the MG groups (both with diplopia and without diplopia) than in the normal group (two-tailed t-test with Bonferroni correction: Ps<0.05; figure 2). In contrast, there were no significant main effects of 20 s fractions or interaction of groups×fractions (*F*_(2, 66)_=1.32, P=0.27 or *F*_(6, 66)_=1.02, P=0.42, respectively; table 1).

**Table 1:** Temporal fixation stability. Values are mean±SD of 68% BCEA (the upper table), the horizontal (X) and vertical (Y) fluctuations (the lower table) in the normal (Normal) patients, patients with MG without diplopia (MG diplopia−), patients with MG with diplopia (MG diplopia+) and SO palsy groups during the first (0–20 s), second (20–40 s) and third (40–60 s) fractions in the fixation period

		Normal (n=21)	MG diplopia− (n=5)	MG diplopia+ (n=5)	SO palsy (n=6)
		68% BCEA (°)^2^			
Time from fixation start	0–20 s	3.21±2.89	3.9±1.78	13.97±8.56	6.67±4.49
	20–40 s	2.14±1.54	4.12±2.67	10.2±3.75	5.92±4.96
	40–60 s	2.02±2.18	4.7±4.19	7.11±7.95	7.06±8.99

		SD (°)							
		X	Y	X	Y	X	Y	X	Y
Time from fixation start	0–20 s	0.49±0.27	1.02±0.62	0.5±0.22	1.22±0.29	1.21±0.61	4.87*±3.16	0.74±0.28	1.58±0.98
	20–40 s	0.41±0.22	0.82±0.34	0.43±0.25	1.59±0.62	0.78±0.4	2.41±0.36	0.69±0.31	1.41±0.64
	40–60 s	0.36±0.19	0.73±0.34	0.47±0.25	1.31±0.67	0.68±0.32	1.65±0.83	0.71±0.22	1.49±1.25

*Two-tailed t*-*test with Bonferroni correction P<0.05 versus the third fraction of MG diplopia+.

BCEA, bivariate contour ellipse area; MG, myasthenia gravis; SO, superior oblique.

**Figure 3:**
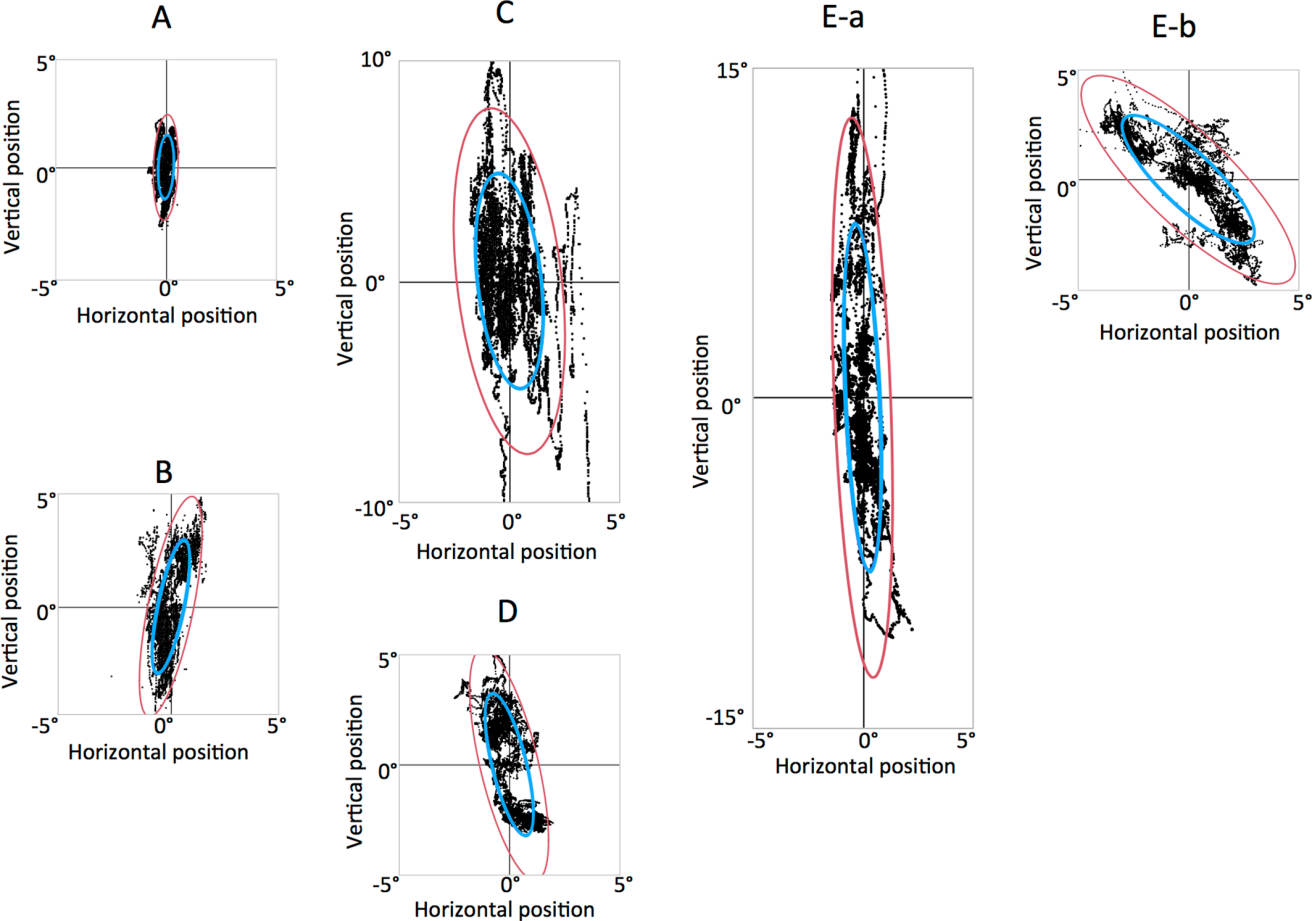
Typical fixation testing results in a normal subject (A), a patient with myasthenia gravis (MG) without diplopia (B), a patient with MG with diplopia (C) and a patient with superior oblique (SO) palsy (D). Results are also compared in a patient with MG before (E-a) and 2 months after (E-b) initiating MG treatment. Each dot on all plots represents eye position, as measured by the eye tracker. Blue and red ellipses represent the 68% and 95%, respectively, of the bivariate contour ellipse areas (BCEA) encompassing the fixation points.

Typical fixation scatter plots are shown for each group in figure 3. By observation, the variation in the normal group was smaller (figure 3A) than in all other groups (figure 3B–E). Individual variation was markedly greater in the MG group (figure 3B,C) than in other groups, particularly in the vertical direction. A patient with MG with diplopia was examined before and after MG treatment, which was accompanied with amelioration of the disease state as observed in the clinical symptoms, such as diplopia and ptosis. After the treatment, the BCEAs were markedly decreased from those of the pretreatment values (figure 3E).

### Upward gaze stability based on the measure of vertical and horizontal SDs

A repeated measures ANOVA test revealed that there was a significant main effect of study groups for SD in both the X and Y eye positions (for X, *F*_(3, 33)_=8.90, P=0.0002; for Y, *F*_(3, 33)_=17.1, P<0.0001). The SD of X position was significantly larger in the MG with diplopia group than in the normal group (two-tailed t-test with Bonferroni correction, P<0.05; figure 2). The SD of Y position was significantly larger in the MG with diplopia group than the other groups including the MG without diplopia group (two-tailed t-test with Bonferroni correction, all Ps<0.05); the SD of Y position in the MG without diplopia group was also significantly larger than the normal group (two-tailed t-test with Bonferroni correction, P<0.05; figure 2).

For the SDs of X position, there was a main effect of fractions (*F*_(2, 66)_=4.81, P=0.011) but no interaction of groups×fractions (*F*_(6, 66)_=1.64, P=0.15; table 1). For the SD of Y eye position, there were both main effect of fractions and interaction of groups×fractions (*F*_(2, 66)_=7.88, P=0.0009 and *F*_(6, 66)_=5.52, P=0.0001, respectively; table 1). The SD of Y eye position in the MG with diplopia group was significantly larger during the first 20 s period than that in the third 20 s period (two-tailed t-test with Bonferroni correction, P<0.05; table 1).

## Discussion

### Directional selectivity of fixation stability during the upward gaze

This study showed that fixation fluctuation during the upward gaze was larger in the vertical direction than in the horizontal direction in all subjects. The upper gaze was used in the current study because it has been shown to be suitable for detecting the MG fatigue phenomenon.^6^ To the best of our knowledge, this is the first study to examine upward gaze stability in healthy subjects and patients with MG. Therefore, the difference in fixation stability between the vertical and horizontal directions observed in the present study could be related to the direction of the gaze, although, to get solid evidence for this relation, additional tests using gazes in left and right directions are necessary. The directional selectivity of fixation stability observed in the present study may stem from the peripheral mechanism needed to maintain the upward gaze. Anatomical factors could play a role: muscle bulk is greater for the superior rectus (SR) muscle than for the other recti^21^; the SR muscle load is greater than for the other vertically acting muscle in rabbits^22^; the SR has fewer muscle spindles than the other recti.^23^ During upward fixation, the SR and the inferior oblique (IO) contract. Persistent contraction of the SR and the IO may produce fatigue in these extraocular muscles, which could lead to larger fluctuation of eye position in the vertical direction. Indeed, this speculation is consistent with the present observation that the fluctuation in vertical direction was much larger in patients with MG than normal subjects.

### Fixation instability in subjects with MG

The present study showed that the upward gaze in patients with MG was more unstable in the vertical position than in normal subjects or patients with SO palsy. Vertical position instability was especially high in the MG with diplopia group. Cleary *et al* have demonstrated that in patients with MG, weakness of the elevator muscles (ie, the SR and the IO) is significantly greater than that of other extraocular muscles.^15^ Almog *et al* also showed that the IO was involved more often than any other extraocular muscle in patients with MG.^16^ Such a trend of extraocular muscle dysfunction (more severe and frequent impairment of the elevator muscles) in patients with MG, which was the reason for using upward gaze in the present testing, may underlie the fixation instability of patients with MG in upward gaze.

In spite of the well-known ocular symptoms of patients with MG, no reports on gaze stability in patients with MG were identified in the academic literature. However, some studies that evaluated central fixation stability using BCEAs in patients with amblyopia and macular degeneration found that visual fixation was unstable in eyes with visual impairments.^8 10 20^ Patients with strabismus have unstable fixation in the deviated eye.^24^ Diplopia may also have unstable fixation because of retinal image fusion attempts. However, diplopia is not the underlying cause of the fixation instability observed here in MG subjects because SO palsy subjects (who also had diplopia) did not have the same degree of instability. In addition, the fixation in patients with MG without diplopia was more stable than that in patients with MG with diplopia, but not similar to normal subject. Therefore, it is likely that the marked fixation fluctuation in MG subjects with diplopia was caused by MG fatigability and not by diplopia.

The present study showed no significant changes in BCEA and SD of Y over time (determined by comparing three consecutive 20 s periods) in normal subjects, in MG subjects without diplopia and in SO palsy subjects. In contrast, patients with MG with diplopia had a tendency to show increased BCEA and SD of Y during the first 20 s period in MG subjects with diplopia. This result was surprising because in the present study it was initially expected that easily fatigued muscles in MG subjects would cause fixation to become more unstable over time. Not yet fully understood, but it may be that extraocular muscles, which at least partially functioned during the first 20 s period, attempted to correct eye position drift towards the upward gaze. However, as muscles became fatigued, ocular position corrections would have become smaller and less frequent, resulting in a smaller fluctuation of eye positions during the second and third 20 s periods compared with those during the first 20 s period. That is, the unexpected result may have been caused by the decrease in eye position correction ability of the patients with MG with diplopia along with the lapse of time. In support of this theory, Feldon *et al* have reported that patients with MG exhibit commonly substantial instability of eye positions immediately after saccadic movements (they called this ‘wavering fixation’).^25^ They commented that wavering fixation was the expression of varying levels of tonic innervation to agonist and antagonist resulting from fatigue that caused the eye to drift. Also, in the present study, the vertical eye position in some of the participants of MG group drifted from the target position (data not shown). Furthermore, the vertical SD value of this group further decreased from the second 20 s period (2.41) to the third 20 s period (1.65), although this did not reach the level of the statistical significance. To confirm the present finding, it was necessary to further investigate by increasing the number of cases of MG.

### Usefulness of eye tracking in evaluating fixation stability in patients with MG

Current clinical evaluations of ocular symptoms in patients with MG with diplopia (initial diplopia and residual diplopia following treatment) are subjective. A published report on the diagnosis and evaluation of MG-associated diplopia describes methods for confirming acquired strabismus caused by MG. These include comparing ocular deviation in the Hess screen test before and after edrophonium infusion.^7^ Electromyography demonstrates the fatigue phenomena electrophysiologically and the edrophonium test shows the pharmacological condition of the fatigue phenomenon. However, these tests are diagnostic invasive methods (ie, electromyography is painful^26^; edrophonium has effect on cardiovascular system^27^), and therefore, ophthalmologists avoid repeated use of these tests if alternative, non-invasive methods are available. The ice pack test, which involves cooling the orbital cavity through the application of ice over closed eyelids for 2 min, is less invasive for diagnosing myasthenic ptosis.^28^ In contrast, the ice pack test for diagnosing myasthenic diplopia is more invasive because it requires a 5 min or longer cooling time because extraocular muscles are located in the deeper portion of the orbital cavity.^29 30^ Unfortunately, the ice pack test becomes increasingly uncomfortable for the patient when cooling times are longer than 2 min.^28^ Compared with these clinical tests, the present quantification method using the eye-tracking system can non-invasively evaluate gaze stability in patients with MG almost without discomforts. As an improvement in gaze stability indicates an improvement in the MG disease state,^3–6^ eye tracking is especially suitable for evaluating treatment efficacy in patients with MG.

Although all the patients met the diagnostic criteria of MG^3^ in the present study, not all patients have undergone all tests. This is one of the limitations of this study, making it impossible to further analyse relationships between the degree of fixation instability of the patients with MG and their disease states.

Ocular symptoms associated with MG strongly contribute to the quality of life decline observed in patients with MG.^31^ Therefore, evaluating diplopia is important, even after treatment has been initiated. The currently used Hess screen and APCT measure ocular motility and position at only one time point. Therefore, they do not accurately evaluate fatigability over time. Evaluating gaze stability using an eye-tracking system is beneficial to diagnosis and evaluation of treatment efficacy in patients with MG because it is non-invasive, quantitative and accurately measures eye position over a long enough period of time to evaluate the muscle fatigability that is characteristic of MG.

## References

[1] Murai H, Yamashita N, Watanabe M, et al Characteristics of myasthenia gravis according to onset-age: Japanese nationwide survey. J Neurol Sci 2011;305:97–102. doi:10.1016/j.jns.2011.03.004 21440910 10.1016/j.jns.2011.03.004

[2] Zambelis T, Pappas V, Kokotis P, et al Patients with ocular symptoms referred for electrodiagnosis: how many of them suffer from myasthenia gravis? Acta Neurol Belg 2015;115:671–4. doi:10.1007/s13760-015-0460-x 25822064 10.1007/s13760-015-0460-x

[3] Japonica SN Practical guideline for myasthenia gravis 2014. Tokyo: Nankodo Co., Ltd, 2014.

[4] Jaretzki A, Barohn RJ, Ernstoff RM, et al Myasthenia gravis: recommendations for clinical research standards. Task Force of the Medical Scientific Advisory Board of the Myasthenia Gravis Foundation of America (MGFA). Neurology 2000;55:16–23. 10891897 10.1212/wnl.55.1.16

[5] Burns TM, Conaway MR, Cutter GR, et al Construction of an efficient evaluative instrument for myasthenia gravis: the MG composite. Muscle Nerve 2008;38:1553–62. doi:10.1002/mus.21185 19016543 10.1002/mus.21185

[6] Suzuki S, Komai K, Mimura O, et al Myasthenia gravis in children Upward gaze test. Jpn Rev Clin Ophthalmol 1994;88:458–60.

[7] Coll GE, Demer JL The edrophonium-Hess screen test in the diagnosis of ocular myasthenia gravis. Am J Ophthalmol 1992;114:489–93. doi:10.1016/S0002-9394(14)71863-X 1415462 10.1016/s0002-9394(14)71863-x

[8] Crossland MD, Sims M, Galbraith RF, et al Evaluation of a new quantitative technique to assess the number and extent of preferred retinal loci in macular disease. Vision Res 2004;44:1537–46. doi:10.1016/j.visres.2004.01.006 15126063 10.1016/j.visres.2004.01.006

[9] Di Russo F, Pitzalis S, Spinelli D Fixation stability and saccadic latency in élite shooters. Vision Res 2003;43:1837–45. doi:10.1016/S0042-6989(03)00299-2 12826107 10.1016/s0042-6989(03)00299-2

[10] González EG, Wong AM, Niechwiej-Szwedo E, et al Eye position stability in amblyopia and in normal binocular vision. Invest Ophthalmol Vis Sci 2012;53:5386–94. doi:10.1167/iovs.12-9941 22789926 10.1167/iovs.12-9941

[11] Serra A, Ruff R, Kaminski H, et al Factors contributing to failure of neuromuscular transmission in myasthenia gravis and the special case of the extraocular muscles. Ann N Y Acad Sci 2011;1233:26–33. doi:10.1111/j.1749-6632.2011.06123.x 21950972 10.1111/j.1749-6632.2011.06123.x

[12] Khanna S, Liao K, Kaminski HJ, et al Ocular myasthenia revisited: insights from pseudo-internuclear ophthalmoplegia. J Neurol 2007;254:1569–74. doi:10.1007/s00415-007-0591-y 17713827 10.1007/s00415-007-0591-y

[13] Yee RD, Whitcup SM, Williams IM, et al Saccadic eye movements in myasthenia gravis. Ophthalmology 1987;94:219–25. doi:10.1016/S0161-6420(87)33470-0 3587896 10.1016/s0161-6420(87)33470-0

[14] Tedeschi G, Di Costanzo A, Allocca S, et al Saccadic eye movements analysis in the early diagnosis of myasthenia gravis. Ital J Neurol Sci 1991;12:389–95. doi:10.1007/BF02335779 1791133 10.1007/BF02335779

[15] Cleary M, Williams GJ, Metcalfe RA The pattern of extra-ocular muscle involvement in ocular myasthenia. Strabismus 2008;16:11–8. doi:10.1080/15569520701830992 18306117 10.1080/15569520701830992

[16] Almog Y, Ben-David M, Nemet AY Inferior oblique muscle paresis as a sign of myasthenia gravis. J Clin Neurosci 2016;25:50–3. doi:10.1016/j.jocn.2015.08.026 26531848 10.1016/j.jocn.2015.08.026

[17] Thaler L, Schütz AC, Goodale MA, et al What is the best fixation target? The effect of target shape on stability of fixational eye movements. Vision Res 2013;76:31–42. doi:10.1016/j.visres.2012.10.012 23099046 10.1016/j.visres.2012.10.012

[18] Aguilar C, Castet E Gaze-contingent simulation of retinopathy: some potential pitfalls and remedies. Vision Res 2011;51:997–1012. doi:10.1016/j.visres.2011.02.010 21335024 10.1016/j.visres.2011.02.010

[19] Shneor E, Hochstein S Eye dominance effects in conjunction search. Vision Res 2008;48:1592–602. doi:10.1016/j.visres.2008.04.021 18541282 10.1016/j.visres.2008.04.021

[20] Subramanian V, Jost RM, Birch EE A quantitative study of fixation stability in amblyopia. Invest Ophthalmol Vis Sci 2013;54:1998–2003. doi:10.1167/iovs.12-11054 23372053 10.1167/iovs.12-11054 PMC3604910

[21] Tian S, Nishida Y, Isberg B, et al MRI measurements of normal extraocular muscles and other orbital structures. Graefes Arch Clin Exp Ophthalmol 2000;238:393–404. doi:10.1007/s004170050370 10901470 10.1007/s004170050370

[22] Okano M [Study on mechanical properties of extraocular muscles. I. Passive length-tension curves in rabbits]. Nippon Ganka Gakkai Zasshi 1992;96:295–301. 1580211

[23] Lukas JR, Aigner M, Blumer R, et al Number and distribution of neuromuscular spindles in human extraocular muscles. Invest Ophthalmol Vis Sci 1994;35:4317–27. 8002252

[24] Economides JR, Adams DL, Horton JC Variability of ocular deviation in strabismus. JAMA Ophthalmol 2016;134:63–9. doi:10.1001/jamaophthalmol.2015.4486 26562632 10.1001/jamaophthalmol.2015.4486 PMC4713272

[25] Feldon SE, Stark L, Lehman SL, et al Oculomotor effects of intermittent conduction block in myasthenia gravis and Guillain-Barré syndrome. An oculographic study with computer simulations. Arch Neurol 1982;39:497–503. 6285871 10.1001/archneur.1982.00510200039007

[26] Buchman AS, Garratt M Determining neuromuscular jitter using a monopolar electrode. Muscle Nerve 1992;15:615–9. doi:10.1002/mus.880150513 1316555 10.1002/mus.880150513

[27] Deschamps A, Backman SB, Novak V, et al Effects of the anticholinesterase edrophonium on spectral analysis of heart rate and blood pressure variability in humans. J Pharmacol Exp Ther 2002;300:112–7. doi:10.1124/jpet.300.1.112 11752105 10.1124/jpet.300.1.112

[28] Kearsey C, Fernando P, D’costa D, et al The use of the ice pack test in myasthenia Gravis. JRSM Short Rep 2010;1:1–3. doi:10.1258/shorts.2009.090037 21103106 10.1258/shorts.2009.090037 PMC2984327

[29] Ellis FD, Hoyt CS, Ellis FJ, et al Extraocular muscle responses to orbital cooling (ice test) for ocular myasthenia gravis diagnosis. J Aapos 2000;4:271–81. doi:10.1067/mpa.2000.106204 11040476 10.1067/mpa.2000.106204

[30] Chatzistefanou KI, Kouris T, Iliakis E, et al The ice pack test in the differential diagnosis of myasthenic diplopia. Ophthalmology 2009;116:2236–43. doi:10.1016/j.ophtha.2009.04.039 19744729 10.1016/j.ophtha.2009.04.039

[31] Suzuki S, Murai H, Imai T, et al Quality of life in purely ocular myasthenia in Japan. BMC Neurol 2014;14:142 doi:10.1186/1471-2377-14-142 24996227 10.1186/1471-2377-14-142 PMC4088302

